# Turbidimeter Design and Analysis: A Review on Optical Fiber Sensors for the Measurement of Water Turbidity

**DOI:** 10.3390/s91008311

**Published:** 2009-10-20

**Authors:** Ahmad Fairuz Bin Omar, Mohd Zubir Bin MatJafri

**Affiliations:** School of Physics, University Science Malaysia, 11800 Penang, Malaysia; E-Mail: mjafri@usm.my

**Keywords:** optical fiber sensor, particles, scattered light, turbidimeter, turbidity

## Abstract

Turbidimeters operate based on the optical phenomena that occur when incident light through water body is scattered by the existence of foreign particles which are suspended within it. This review paper elaborates on the standards and factors that may influence the measurement of turbidity. The discussion also focuses on the optical fiber sensor technologies that have been applied within the lab and field environment and have been implemented in the measurement of water turbidity and concentration of particles. This paper also discusses and compares results from three different turbidimeter designs that use various optical components. Mohd Zubir and Bashah and Daraigan have introduced a design which has simple configurations. Omar and MatJafri, on the other hand, have established a new turbidimeter design that makes use of optical fiber cable as the light transferring medium. The application of fiber optic cable to the turbidimeter will present a flexible measurement technique, allowing measurements to be made online. Scattered light measurement through optical fiber cable requires a highly sensitive detector to interpret the scattered light signal. This has made the optical fiber system have higher sensitivity in measuring turbidity compared to the other two simple turbidimeters presented in this paper. Fiber optic sensors provide the potential for increased sensitivity over large concentration ranges. However, many challenges must be examined to develop sensors that can collect reliable turbidity measurements *in situ*.

## 1. Introduction

Turbidity analysis is the study of the optical properties that causes light through water to be scattered and absorbed rather than transmitted in straight lines. Turbidity causes cloudiness or a decrease in transparency of water. The direction of the transmitted light path will undergo changes when the light hits the particles in the water column. If the turbidity level is low, less light will be scattered away from its original direction. Light scattered by particles such as silt, clay, algae, organic matter and microorganisms may enable the detection of these particles in water [1,2]. A turbidimeter or sometimes called as turbiditimeter (turbidity meter) is a common name for an instrument that measures turbidity. Measuring low level turbidity requires an accurate measurement of the scattered light in water [3]. With advances in the development of photo detector sensors, later turbidimeter designs are able to detect very small changes (attenuation) of transmitted light intensity through a fixed volume sample. However, designs still lack of the capability to measure high or very low levels of turbidity. For sample with low turbidities, the scattering intensities will be very small and hard to detect since the signal might be lost in the electronics noise, while for higher turbidities, the existence of multiple scattering will interfere with the direct scattering. There is a method to improve the signal to noise ratio. This technique measures the light scattered at an angle to the incident light. The 90° detection angle is considered to be the most sensitive angle to measure scattered light and it is recognized by EPA (Environmental Protection Agency) Method 180.1 [4]. Generally, there are two main types of turbidimeters [5]. They can be categorized as:

- Absorptiometers: which measure the absorption (or attenuation) of a light intensity passing through the sample.
- Nephelometers: which measure the portion of light scattered at angle 90° from the incident beam.

**Figure 1.**
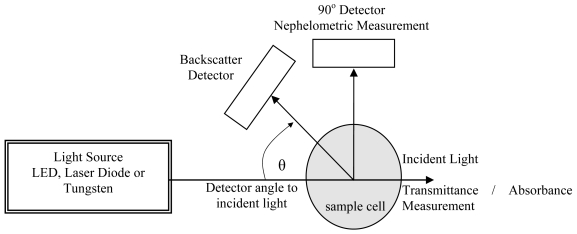
Turbidity Measuring Techniques.

Besides these measurement techniques, backscattering refers to the measurement of scattered light at an angle between 90° to 180°. Figure 1 shows various configurations for measuring turbidity through an optical system [6].

The U.S. Environmental Protection Agency regulations require that municipal wastewater treatment plants must provide treatment to meet total suspended solids (TSS) limits of 30 mg/L at the point of discharge from the treatment facility [7]. Interim National Water Quality Standards (INQWS) state that the acceptable range of TSS for Malaysian rivers is 25 to 50 mg/L and the threshold level of TSS for supporting aquatic life in fresh water ecosystems is 150 mg/L. In addition, according to International standards the acceptable level of turbidity of water for domestic use ranges between 5 to 25 NTU [8]. However, Malaysian Ministry of Health has set a threshold level of low water turbidity at 1,000.00 NTU [8].

This review paper will discuss the possible factors that may affects the measurements of turbidity which comprise of the particles' properties that contribute to water turbidity and the instrumentation properties that covers the optical components and angle of measurement for effectively measuring different levels of turbidity. Besides, this paper also elaborates on the relationship between turbidity, total suspended solids (TSS) and suspended sediment concentration (SSC). Correlations have been demonstrative in pure samples in the laboratory, however, the consistency of these relationships over a range of concentrations and flow velocities in the field has not been demonstrated. Such field measurements will require *in situ* probes that have been carefully designed and calibrated to correct for the many variables that influence turbidity measurements.

## 2. Relationship between Turbidity (NTU) and TSS (mg/L)

There are various parameters which can be associated with water quality. One of the common variables often measured and correlated to water quality is the TSS capacity per unit liter of pure water (mg/L). While in the other hand, water quality can also be represented in its appearance, which relates to its clarity and specifically defined as turbidity with the standard unit of measurement in NTU. In some instances, these two parameters may be correlated. Holliday *et al.*, Daraigan, Omar and MatJafri and Baker *et al.* [9-12] have conducted experiments to show a relationship between turbidity expressed in the NTU unit with the TSS in pure samples (non environmental) in the mg/L unit. Through the experiments (Table 1), it is found that turbidity has a strong relationship with TSS, as stated by Equation 1:

(1) $$\text{NTU}=\text{a}{\left(\text{TSS}\right)}^{\text{b}}$$

where a and b are regression-estimated coefficients and b is approximately equal to one for all particles [9].

However, besides depending on suspended particles, turbidity also relies on many other factors such as the presence of organic matter and other floating debris, algae, air bubbles and water discoloration. Therefore, in this instance, correlation of turbidity measurements with suspended particles can arguably be inconsistent due to the existence of large variability in the signal caused by components other than suspended particles [13,14]. Additionally, the correlation between turbidity and suspended particles usually fails at high concentrations. At this stage, the calibration between turbidity and light scattering becomes non-linear [15]. Situations may be much complicated when the relationship between turbidity and suspended particles is derived at a particular site, such as at agricultural fields where a very high variability of particles compositions exists. To overcome this, the size of drainage or watershed can be reduced to the scale of individual fields or plots to make the soils and contributing areas become less variable [16].

Particle size, configuration, color and refractive index will determine the spatial distribution of the scattered light intensity around the particle which is one of the contributors that determines the relationship between turbidity and suspended particles [3,16]. Particles with sizes much smaller than the wavelength of the incident light will scatter light with roughly equal intensity in all directions. Particles larger than the wavelength of the incident light will create a spectral pattern that results in greater light scattering in the forward direction than in the other directions [17,18]. The intensity and pattern of the light transmitted through the water is also relying on the particles tendency to absorb certain wavelengths of the incident light [17]. Campbell *et al.* [15] have conducted an experiment using a fiber optic in-stream transmissometer to observe the influence of particles' color in the measurement of light transmission. The relationship between particle concentration and the reciprocal of light transmission is found to be linear in pure (non environmental) samples. The R^2^ for the trend lines were found to be 0.973, 0.988 and 0.994 for pale yellow sand, brown core sample and light olive brown channel particles, respectively. Slopes of the graph on the other hand ranged from 0.0858 to 0.0968. However, Campbell *et al.* also argued that the differences observed are partly due to the difference geometries of the particles. The experiment was conducted on particles from the same size class (150–200 μm). This statement can be further clarified through particles analysis conducted by Jury *et al.* and Sparks [19-20]. According to them, clay particles are made up of illite, montmorillonite, kaolinite, halloysite and commonly shaped as plates, disks and fibers. Quartz sands appear more spherical and with a greater width.

Particles with smaller size have a tendency to settle down much slower compared to those with larger size [9]. This scenario will affect the measurement of turbidity when similar samples are measured at different times. The terminal settling velocity is calculated through the drag, buoyant and gravitational forces acting on the particle [21]. Particle settling or sedimentation can be explained through the Newton equation for terminal settling velocity of a spherical particle. The rate for discrete particles to settle in a fluid at constant temperature is given by the Equation 2 [21]:

(2) $$V={\left[(4g(\rho_{s}-\rho )d)/(3C_{d}\rho )\right]}^{0.5}$$

where:

V = terminal settling velocity
g = gravitational constant
ρs = mass density of the particle
ρ = mass density of the fluid
d = particle diameter
Cd = Coefficient of drag (dimensionless)

The following is the example of time taken for several solids for its sedimentation [22].

- Clay <2 μm − 14 days to sink 5 cm water.
- Silt 2–20 μm − 3.5 days.
- Sand 20–2,000 μm − 1.5 seconds.

Frequently, geologists and soil scientists will define clay as a particle with a size less than 2 μm, while sedimentologists will use 4 μm and colloid chemists use 1 μm. Sedimentologists may use the term “*clay*” to generally refer to grain size. However, it is more accurate to give the actual dimensions of the particles. ISO 14688 grades clay particles as being smaller than 2 μm, silts between 2 μm and 63 μm and sand between 63 μm and 2,000 μm [23,24].

## 3. Light Scattering Phenomena

When light is transmitted onto a water body, the suspended particles will block the transmission of light from going through the water. In pure or very clear water, the light transmission will be largely uninterrupted, with a small scattering effect. The pattern of interaction between light and suspended solids is depending on the size, shape and composition of the particles in the solution and to the wavelength of the incident light. Besides the scattering effect, the transmitted light will also be absorbed and attenuated in its intensity by the particles [4]. Therefore, the equation can be derived based on Beer-Lambert law as shown by Equation 3 [25-27]:

(3) $$\text{I}={\text{I}}_{\text{o}}{\text{e}}^{-\left[\alpha \text{a}+\alpha \text{b}\right]\text{xc}}$$

where

I = resultant light intensity
I_0_ = light intensity at point 0.
0 = starting point of the light passage through the absorbing medium.
X = length of the medium or the distance of light travel through the medium.
c = since the medium is a solution, the concentration is included
αa = absorption coefficient.
αb = scattering coefficient.

If the solution consists of particles with different absorbing and scattering coefficients, the total absorption and scattering coefficient, αa and αs, are equal to the sum of the absorption and scattering coefficients of all the particles [26].

Water can account for approximately 80% of backscattering in the blue part of the spectrum in the clearest waters [28,29]. The contribution of water to backscattering varies spectrally, decreasing with approximately the forth power of wavelength [30]. Besides, the scattering occurs at small angles from the original path, to the side or backwards with about 1.5 percent at angles greater than 90° [25]. The following are the absorption and scattering coefficient produced by pure water, measured by Pope *et al.* and Buitveld *et al.* [31,32] respectively.

- Absorption Coefficient (λ_470nm_) = 0.0130 m^−1^
- Absorption Coefficient (λ_635nm_) = 0.3309 m^−1^
- Scattering Coefficient (λ_470nm_) = 0.0027 m^−1^
- Scattering Coefficient (λ_635nm_) = 0.0008 m^−1^

Total scattering coefficient can be divided into forward scattering (scattering between angle 0° and 90°) and backscattering (scattering between angle 90° and 180°) and be represented by the Equations 4 and 5 [33-35]:

(4) $$b_{f}=2\pi \int_{0}^{\pi /2}\beta (\theta )\sin\theta d\theta [m^{-1}]$$

(5) $$b_{b}=2\pi \int_{\pi /2}^{\pi }\beta (\theta )\sin\theta d\theta [m^{-1}]$$

Therefore, the total scattering coefficient, b, can be represented as:

(6) $$b=b_{f}+b_{b}$$

Where β(θ) is the volume scattering function which by definition is “*the fraction of energy scattered from a collimated beam at a particular angle θ from a given scattering volume*” [12].

**Figure 2.**
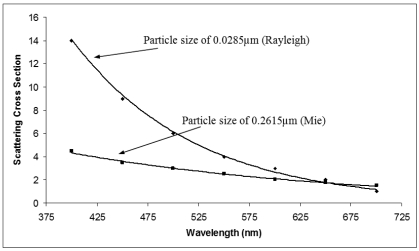
Rayleigh scattering and Mie scattering cross section vs wavelength for particle size of 0.0285 μm and 0.2615 μm, respectively [31].

Cox *et al.* [36] have conducted an experiment using a conventional absorption spectrophotometer to measure the total light scattering cross sections for small particles throughout the visible spectrum. Figure 2 shows a graph of the results attained by Cox *et al.* to represent Rayleigh and Mie scattering cross section against visible light wavelength. From the graph, there is an indication that Mie scattering is less dependent on the wavelength if compared to Rayleigh scattering. If the light incident on large particles is white in color, the particles are capable to scatter all wavelengths of white light equally. While, in the other hand, smaller particles tend to scatter the shorter wavelengths of white light such as violet, blue and green more effectively than the longer orange, yellow and red wavelengths.

For the backscattering measurement techniques (90° < θ < 180°), the identification of scattering angle that produces the best scattering coefficient is desirable in order to acquire the highest intensity of scattered light. According to Oishi, measurements of 120° provide a good proxy for the backscattering coefficient. However, Dana and Maffione argued that measuring 140° provides a good proxy b_b_ as well [37]. Daraigan [10] has conducted an experiment to identify the angle of measurement of scattered (forward and backscattered) light that produces the best R^2^ with the lowest RMSE. The result of the experiment is shown in Table 2. It is identified from this experiment that 90° measurement techniques provide the best R^2^ with lowest RMSE, which is also angle of measurement specified by EPA Method 180.1.

## 4. Light Sources and Detectors

EPA Method 180.1 has specified that tungsten lamp, with a color temperature of 2,200–3,000 K shall be used as the light source for turbidimeter and this is in fact the most common light source used. The band of light wavelengths generated by a lamp, also known as its spectral output, is usually characterized by its “*color temperature*” which is the black body radiator temperature required to produce a certain color. The tungsten filament lamps are incandescent lamps and have a wide spectral band that contains many different wavelengths of colors. However, the production of numerous wavelengths of light by the tungsten filament lamp can lead to the lower intensity of the scattered light. This is due to the fact that natural color and natural organic matter in the sample can absorb some specific wavelengths [3]. In order to overcome some of the incandescent lamp limitations, some turbidimeter designs use monochromatic light sources [3]. A light emitting diode (LED) with a wavelength of 860 nm and a spectral bandwidth less than or equal to 60 nm is specified by the ISO 7027 as the light source [38]. The presence of the dissolved color in the sample may affect the reading of turbidimeter. However, the use of a light source at this wavelength can minimize this constraint, since near infrared light source is less influenced by the color of the sample [5]. Monochromatic light has a very narrow band of light wavelengths which make them only having a few colors. By this way, selection of light wavelengths that are not normally absorbed by organic matter can be done so the light will be less susceptible to interference by sample color. However, some of these different light sources respond differently to particle size and are not as sensitive to small sized particles if compared to the tungsten filament lamp [3].

In turbidimeters, the light produced from the interaction of the incident light and the sample volume will be detected by the photodetectors and as a result the electronics signal produced is then converted to a turbidity value. The location of the detector in the turbidimeter varies according to the design configuration of the instrument. There are four common detectors used in turbidimeter, including photomultiplier tubes, vacuum photodiodes, silicon photodiodes, and cadmium sulfide photoconductors [17]. Each of the four detectors stated above respond differently to certain wavelengths of light. When a monochromatic light source is used, the specification of the photo detector is not nearly as critical. Generally, when the polychromatic tungsten filament lamp is used as a light source, the photomultiplier tube and the vacuum photodiode are more suitable as a detector. This is because the sensors are more sensitive to the shorter wavelength light in the source. This design configuration is more sensitive to the detection of smaller particles. On the other hand, the silicon photodiode is more sensitive to longer wavelengths in the light source and making it more suitable to be used for sensing larger particles. Cadmium sulfide photoconductor sensitivity is in between photomultiplier tube and the silicon photodiode [3].

## 5. Tolerance in Design

Even though various types of instruments have been produced through technological advances in the water turbidity measurement, different designs of turbidity instruments do not always lead to identical results. Furthermore, there are many factors that can contribute to the measurement of turbidity. These include the color of dissolved constituents in the water matrix and particulate materials, particle size and density. The combination between different sources of waters from various environmental samples may not produce a linear result when measuring turbidity [39]. Differences in optical design of nephelometers such as spectral emission of light source, spectral sensitivity of detector, angular range of detector and beam configuration may contribute in varying the measured result [25]. The deviations in reading turbidity values may still occur even when all different instruments are calibrated using formazin, the intensely scattering suspension used as the standard in nephelometry. Therefore, several practices need to be committed in order to ensure the accuracy of defining the value of turbidity from different instrumentation:

- Report turbidity on the basis of the individual instrument design.
- Use identically prepared calibration solutions.
- Use consistent techniques and instrumentation throughout a data-collection program.

Besides, sensor fouling such as scratches on the surface of the optical detector will produce a wrong measurement that will either increase or decrease the intensity of scattered light onto the sensor surface. Likewise, the existence of bubbles or gases in the water can also disturb the actual reading of turbidity [39].

## 6. Optical Fiber Sensor

In general, an optical fiber sensory system consists of a light source, optical fiber; a sensing element (transducer) and a detector. The operating principle of a fiber based sensory system is that the transducer modulates some parameter of the optical system such as intensity, wavelength, polarization or phase of light signal. This will gives rise to a change in the characteristics of the optical signal received at the detector. The fiber sensor can be either in intrinsic or extrinsic form. Intrinsic means that the modulation of signal takes place directly in the fiber while for the extrinsic, the modulation is performed by some external transducer [40]. Fiber optic technology presents many degrees of freedom and some advantages such as [40,41]:

- no moving parts
- absolute measurement
- stability (immunity to electromagnetic interference)
- excellent resolution and range
- passive operation, intrinsically safe
- water and corrosion resistant
- compactness (rugged, small size and light weight)
- multiplexed in parallel or in series
- modest cost per channel

In one design and application of optical fiber sensor, Borecki [42] have developed an intelligent fiber optic sensor for estimating the concentration of a solution. The sensor operates based on a stepwise measuring procedure which includes sensor's head submerging, submersion, emerging and emergence from the examined solution [43]. The measured signal will rely on the surface tension, viscosity, turbidity and refraction coefficient of the solution. The deviation of the amplitude of the measured signal against time offers information about the type of liquid [42]. Fiber optics have also been widely applied as a low cost strain sensor for structural monitoring. In this application however, the fiber has undergone some modification to make it have higher sensitivity for the specified application. For instance, fiber Bragg grating (FBG) sensors which comprise an optical fiber with diffraction gratings incorporated into its core. The passage of light through this type of optical fiber will be affected by stretching it. Existing electrical strain gauges that serve for the same purposes suffer from sensitivity to electromagnetic interference, whereas FBG sensors do not. Despite of having an excellent sensitivity and versatility, FBG is comparatively expensive and not very mechanically robust [44]. In addition to the examples given, optical fiber sensors have also been applied as interferometric sensors in which the output beam of the sensing waveguide interferes with a reference beam. There are a well established sensors based on interference concept such as Mach-Zehnder interferometers, Young interferometer and Michelson. Besides, resonator type sensors are also relying on interference such as ring resonator and Fabry-Perot resonator. These classes of sensors have a typical resolution in the order of 10^−7^–10^−5^ refractive index units (RIUs) [45]. Thus, the technology of fiber optic system is continuously emerging especially in upgrades to configuration and the materials used in adding its sensitivity particularly for a specific sensory application.

## 7. Relationship between TSS (mg/L) and SSC (mg/L)

The measurement of suspended particles in water column can be performed either in laboratory with proper sampling and stagnant water conditions or also in the field, where particles now are moving together with the flowing medium. Different techniques of measurement have been introduced for application in different environments. Besides, the terminology in defining “*particles*” in both environments may also differ. There is often a tendency among researchers to interchangeably use the term total suspended solids (TSS) and suspended sediment concentration (SSC) in defining particles that are suspended in water column.

The TSS method was originally designed for analyses of wastewater samples. However, TSS method produces unreliable for the analysis of natural water samples. Quite the opposite, the SSC method produces relatively reliable results for samples of natural water. This is true regardless of the amount or percentage of sand-size material in the samples [46]. The primary difference between TSS and SSC are in the preparation of the sample for subsequent filtering, drying, and weighing. TSS analysis generally entails withdrawal of an aliquot of the original sample for subsequent analysis. However, it is reported that there may be a lack of consistency in methods used in the sample preparation phase of the TSS analyses. In the other hand, the SSC analytical method uses the entire water-sediment mixture to calculate SSC values. If a sample contains a significant percentage of sand-size material, stirring, shaking, or agitating the sample before obtaining a subsample will rarely produce an aliquot representative of the sediment concentration and particle-size distribution of the original sample. This is a by-product of the relatively rapid settling properties of sand-size material, compared to those for silt- and clay-size material [46].

Guo [47] has conducted a study on the relationship between TSS, SSC and true concentration of particles with different size ranges (0–8 μm, 8–53 μm, 53–106 μm, 106–250 μm, 250–500 μm, 500–1,000 μm). The SSC concentration was found to always be very close to the true concentration, regardless the concentration and particle size range. For very fine particles (0–8 μm), TSS and SSC were both well correlated and very close with the true concentration. The difference was less than 4%. For fine particles (8–53 μm), TSS and SSC, were well correlated with the true concentration with slightly smaller or moderately smaller than the true concentration, depending on the size of particles. However, when the particle size increases, the correlation between TSS and true concentration starts to fall further. The correlation between TSS with true concentration and SSC fails for particles sizes larger than 106 μm.

## 8. Continuous Monitoring of Particle Concentration in Flowing Media

There are many technologies currently available for measuring suspended particles in flowing media such as flow proportional samplers, sampling pumps, laser diffraction, optical backscattering devices, and acoustic backscattering devices [48]. Optical backscattering (OBS) and acoustic backscattering (ABS) are the most reliable for providing real time measurements. These two techniques have the required temporal resolution in measuring suspended particles in stream or storm runoff [18]. Flow proportional samplers and sampling pumps techniques are time consuming and do not provide a real-time measurement while laser diffraction techniques require a relatively large power source and are expensive [18]. Measurement through optical and acoustic backscattering however does not produce the true value of suspended particles in the water column since the measured value is in turbidity and can be influenced by some other factors such as organic matter and other floating debris, algae, air bubbles and water discoloration [18]. Optical fiber sensors are a promising techniques in remotely and continuously monitoring particle concentration and size in flowing media. Optical fiber sensors are often designed for measuring suspended particles in a transparent medium through backscattered light intensity. Detecting the backscattered light in a medium with suspended particles is suitable only for high concentrations of suspended particles or with relatively small particle sizes. Suspended sediment concentrations (SSC) in urban runoff during large storms can be in excess of 10 g/L [15]. Optical backscattering (OBS) is normally used for measurement of low concentration of suspended sediment in marine applications with lower lateral flow velocities [18]. Turbidity probes based on OBS at 90° and 180° and laser diffraction are the most readily commercial products available for continuous monitoring technologies [15].

**Figure 3.**
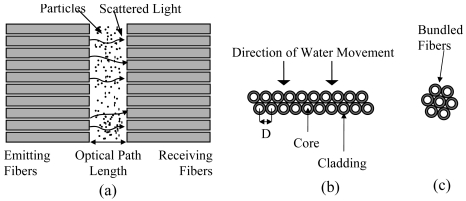
Configurations for (a) PCOS and FIT Experimental Setup (b) Two Adjacent Linear Fibers Layers (c) Bundled Fibers [15,18].

Tran *et al.* [18] have develop a particle concentration optical fiber sensor (PCOS) based on the total light transmittance between a light source and detectors through Monte Carlo simulation. The PCOS arrangement, shown in Figure 3a,b, consists of two linear arrays of ten fibers for both the light receiving and the light emission sides. One end of each fiber on the emission side is illuminated by LED source with wavelength of 472 nm. The other end of fibers illuminates the medium. On the receiving side, it is separated from the emission side by a gap which fluids with suspended particles flows. One end of each fiber collects transmittance light and delivers it to a photo detector which is connected to the other end of fibers. There are two common configurations of fiber bundle which are linear (as shown in Figure 3a,b) and circular (as shown in Figure 3c). The linear packaging that contains the same number of optical fibers is much better for real-time and continuous measurement of flowing media. The linear arrangement of fibers will increase the spatial scanning of particle distribution in a flowing medium which will then increases the spatial–temporal sensitivity of the sensor due to larger crossing lengths. However, this may not be accurate for a stagnant medium. In addition, for the linear fibers arrangement, when a sharp change in particulate concentration flows through the system, all the fibers will receive the change at the same time. On the contrary, for the circular bundled arrangement, the optical fibers encounter the concentration front at different moments. This happen because the sediment front does not reach all the fibers at exactly the same time [15]. Design with two adjacent layers in alternate positions will further improve the spatial scanning of particle distribution [18].

The simulation was tested on different particles sizes (200, 150, 100, 50 and 36 μm). From the results, it is shown that particles with larger diameter scattered light in the forward direction (towards the receiving fibers) at higher intensity. The intensity of detected light reduced accordingly based on the particles diameter sizes. Besides, as the optical path length increases, the graph of detected light intensity becomes more curvilinear and approaches saturation at a lower volume fraction [18].

In another similar design, Campbell *et al.* [15] have constructed fiber optic in-stream transmissometers (FIT) which are also based on the total light transmittance between a paired light source and detector. The purpose of the design is to continuously measure high concentrations of suspended sediments. The light source used in the design is LED with bandwidth range from 603 to 672 nm and a peak at 640 nm. A red LED was selected since it able to reduce any interference that caused by color and focuses on the suspended sediment measurement target. Besides, according to Holdaway *et al.* [49], wavelength around 645 nm is common for suspended sediment measurements. A tunable laser light source with higher quality and stable source was also tested. However, there was no added advantage recorded over the much cheaper LED. Two different detectors were used which are a portable USB2000 spectrometer by Ocean Optics Inc. and a photo detector connected to a Fluke multimeter [15]. This experiment was conducted within a laboratory environment.

## 9. Simple Turbidimeter Design

**Figure 4.**
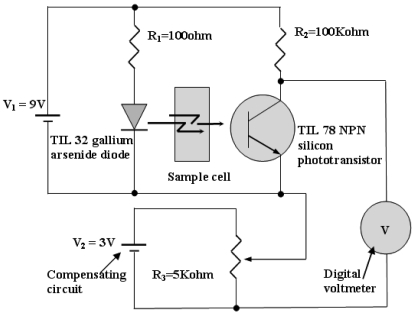
Schematic design of a simple turbidimeter [50].

There are a variety of sensory systems that have been developed for water quality measurement. Some of these systems have a standard design that makes them have a marketable value. Even so, turbidimeters can also be constructed from a very simple arrangement of optical and electronics components. This fact opens up an opportunity for elementary science school students to also use their understanding of fundamental electronics and optical physics for the development of a simple sensory system with a specific application. Mohd Zubir and Bashah [50], students from Al-Mashoor High School, Penang (Malaysia) have constructed a simple sensor for water pollution measurement. They have used simple electronics devices to construct a turbidimeter based on transmittance measurement technique that able to measure TSS in the unit of mg/L. The circuit was designed using near infrared light source (Part No. TIL 32) with peak emission wavelength at 940 nm with a phototransistor detector, TIL78 with responsivity that matches the light source. Figure 4 shows the entire design of the circuit. The result of measurement is indicated by the voltmeter.

**Figure 5.**
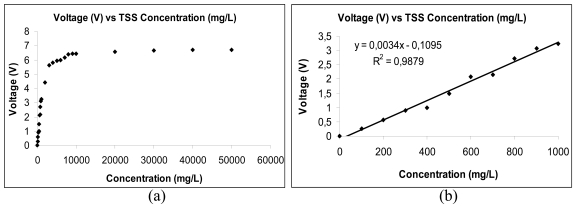
(a) and (b). Results from the experiment conducted by Mohd Zubir and Bashah [50].

The measurement results are shown in Figure 5a,b. Overall data was gathered at range of TSS from 0 mg/L until 50,000 mg/L, as shown in Figure 5a. The segmentized data from low level turbidity was taken from 0 mg/L until 1,000 mg/L, as shown in Figure 5b, where a good linear correlation coefficient, R^2^ = 0.9879 with Root Mean Square Error, RMSE = 38,40238 is obtained between the concentrations of TSS with the measured voltage [50]. In another turbidimeter design and implementation, Daraigan [10] has conducted an experiment for the measurement of TSS in pure water through a newly developed simple multispectral optical system.

**Figure 6.**
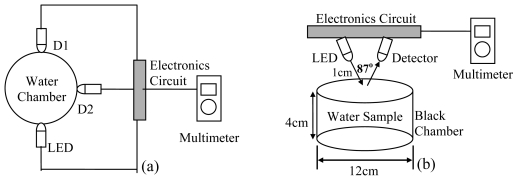
(a) Experimental setup for the measurement of light transmittance and 90° scattering; (b) Experimental setup for the measurement of surface reflectance [10].

TSS samples used in experiments conducted by Mohd Zubir and Bashah [50] and Daraigan [10] were prepared in the laboratory from a combination of clay and silt, fine enough to form a homogenous mixture with water. The supporting circuitry was in a structure similar to that in Figure 4. The experimental setups are shown in Figure 6a,b. However, different optical components and parameters have been used. Three LEDs have been used in the setup. Those LEDs having peak wavelength emission at 950nm (Part No. TSUS520), 875nm (Part No. HSDL4230) and 635nm (Part No. HLMPC115). The detector used in this experiment is a phototransistor, TIL81.

Table 3 shows the entire result of the measurement using the optical components as stated above. Some of the experiments used two or three light sources simultaneously, to produce a multispectral light source to the sample. Through this design and experimental point of view, there is no clear indication which wavelength or configuration that can consistently give the best measurement of turbidity. However, the lowest RMSE and the best linear correlation coefficient (R^2^) recorded was at wavelength of 950 nm using surface reflectance measurement configuration [10]

## 10. Water Quality Fiber Sensor

In the development of water quality fiber sensor, plastic optical fibers with a core diameter of 1 mm were used as the light transferring medium between the sample and the optical system. Due to the very small size of fiber diameter, the coupling between fiber cables to the detector is very crucial. Therefore, a highly sensitive detector is required so that the small resolution of turbidity can be registered by the system [51]. The application of fiber optic cables will introduce a flexible interface between the spectroscopic sensory system and the sample to be examined *in-situ* [52]. Plastic optical fiber cable has a plastic core and cladding. Plastic fiber has a few advantages over glass fiber. It is more flexible, can be bent at 90° with no reduction of light transmission and is more rugged than glass. Plastic fiber is easy to install and can better withstand stress. Besides, it's weigh 60% less than glass. However, since it has the highest attenuation, plastic fiber is most suitable to be used for short distances [53].

**Figure 7.**
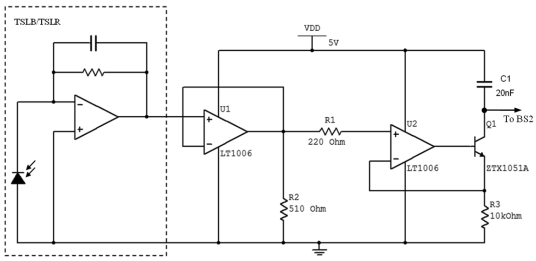
Detector Circuit Design [51].

The optical fiber sensor for water quality measurement developed by Omar and MatJafri [51] consist of two emitter and detector systems that are specifically designed to measure water turbidity level in mg/L units. However, the system can be recalibrated for the measurement in NTU units, which are the standard units for turbidity. The system developed has peak responsivity at 470 nm (BLUE System) and 635nm (RED System). BLUE System consists of LED (Part No. E1L51-3B0A2-02) with typical wavelength of 470 nm and light sensor (Part No. TSLB257) with a peak response at 470 nm. RED System consists of LED (Part No. HLMP-EG08-WZ000) with typical wavelength of 635 nm and light sensor (Part No. TSLR257) with peak response at 635 nm. The TSLB257 and TSLR257 are the light-to-voltage optical converters that combine a photodiode and a transimpedance amplifier on a single CMOS IC. These sensors are integrated with onboard blue and red optical filters respectively. The supporting circuitries for both systems are identical. The comparison between light source and photo detector parameters used in the design of water quality fiber sensor is tabulated in Table 4. RED System demonstrated a higher intensity of LED and a better sensitivity of detector. The detection of scattered light was then submitted to Basic Stamp 2 (BS2) microcontroller for data interpretation and display. Figure 7 shows the detector circuit design with the output pin (voltage) from the photo detector that is connected to the input of the amplification circuit.

**Figure 8.**
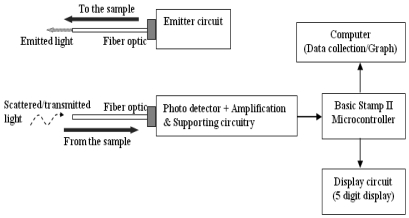
Illustration of Overall Design Concept [54].

The conceptual design of the overall system is illustrated in Figure 8. For every turbid water sample, the TSS (total suspended solids) capacity in the water will be represented by the amount of light received by the detector. The result will be represented in the unit of RCTIME. RCTIME is the discharging time of the capacitor (C1) connected to the BS2 microcontroller. When the BS2 is executed, it will measure the discharging time of the capacitor from 5 V to 1.5 V (changing of binary state from 1 to 0). The RCTIME recorded by BS2 is inversely proportional to the amount of light collected by the detector.

**Figure 9.**
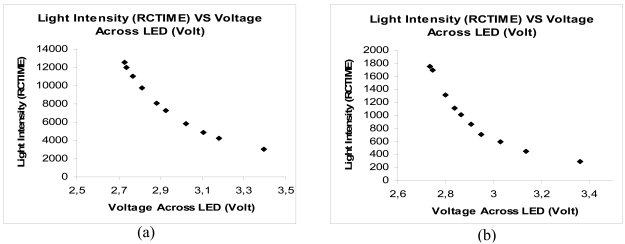
System Response Test for (a) BLUE System (b) RED System.

Figure 9 shows BLUE and RED System response towards the intensity of light which is represented by the amount of voltage applied across white LED and 1 kΩ resistance which are connected in series to each other. Both systems have shown an exponential response towards the intensity of light emitted on the surface of the sensor through a plastic optical fiber cable. The system can best be represented by exponential equation with exponential regression coefficient, R^2^ as shown in Table 5. A higher value of RCTIME is registered by BS2, indicating that a lower intensity of light is detected by the photo detector. Higher voltage applied on LED (represented by x-axis) shows the higher intensity of light is emitted by the LED.

**Figure 10.**
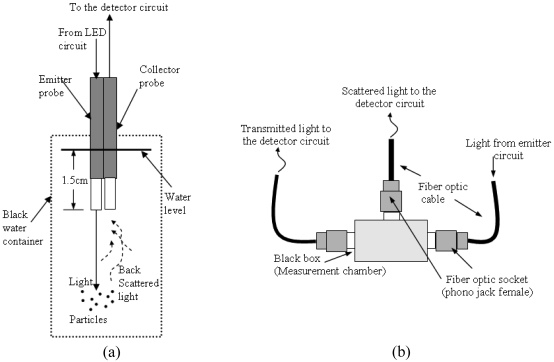
Experiment Setup for (a) Backscattered Light Measurement (b) Transmittance and 90° Light Measurement [51,54].

In the set of experiments conducted using the optical fiber sensor, three different measurement configurations have been developed. The first one is the backscattering measurement (as shown in Figure 10a), second is transmittance measurement (0° scattering) and the third is 90° scattering measurement (as shown in Figure 10b). A black cylindrical plastic water container, with 33 mL volume was used for the backscattered measurement experiment. Only a black container can produce a consistent reading because it minimizes the reading error due to different reflective and refractive indexes when the orientation of the container in the measurement chamber is changed. For transmittance and 90° measurement, a transparent cylindrical plastic water container, with a maximum volume of 20 mL was used. The container was ensured to be free from any scratches, drip of water or foreign substance at the surface of its body. The body of the container was kept free from any fingerprints when the experiment was conducted. It was also ensured that the container is positioned at the same orientation when it is inserted in the measurement chamber. The TSS samples used to generate the calibration equation in all experiments were prepared in the laboratory from a very fine clay sample (brown-dark greenish in color). The TSS was ensured to be fine enough to homogenously mix with reverse osmosis water. Reverse osmosis water was used in the preparation of all samples and was assumed to have 0 mg/L of suspended particles.

In the backscattering measurement experiment, the first set of measurements were conducted to generate the calibration equation for the RED and BLUE Systems. Two consecutive experiments were then conducted using two samples which were taken from the Terengganu River (brown-dark greenish clay) and House Water Supply (orange-rusty looking water). This was done to validate the calibration equation and to test on the ability of the system in measuring samples with different natures and properties while maintaining consistent and reliable results. The relationship between estimated RCTIME (x-axis: initially obtained from the calibration equation) and measured RCTIME (Y-axis: new measurement) were computed for TSS sample between (0–70 mg/L). For transmittance and 90° scattering measurement, the first experiment was also conducted to generate the calibration equation for the RED and BLUE Systems [11,51,54]. In addition to that, for the transmittance configuration, the systems have also gone through a calibration process for the measurement of turbidity in NTU units by using a Lovibond PCCHECKIT Turbidity Meter. This was conducted by Othman *et al.* [55] using samples taken from various rivers in Endau Rompin National Park. The complete results of measurement are summarized in Table 6. From here, it is clearly seen that the best configuration for the measurement of turbidity is at 90° from the incident light. This is in line with the EPA standard for turbidimeter. From R^2^ and RMSE stand point, for backscattering measurement, BLUE System has shown a better result compared to RED System while for transmittance and 90° scattering, RED System has given a slightly better result in the measurement of turbidity.

## 11. Analysis

In the development of optical instruments for the measurement of water turbidity, there are technical factors which need to be considered to ensure that the developed instrument is capable of giving optimum measurement results. These factors includes the selection of the optical parameters for the instrument, including the specification of the wavelength and intensity of the light source and the sensitivity of the detector, which refer to the peak response wavelength and the smallest amount of photon the detector manage to interpret [56]. This is very important since the resolution of the developed turbidimeter is highly dependent on this design aspect. The angle of measurement, between the incident light to the water and the location of the detector does produce a noticeable effect to the resolution of measured turbidity, as well as the intensity of scattered light. This is another important factor to be determined for the implementation of back scattered measurement where the highest detection of signal is desirable. Experiments conducted by Daraigan [10] and Omar and MatJafri [54] determined that 90° measuring technique able to give the best reading of turbidity. For the highest intensity of backscattered light measurement, Oishi, claimed that it occurs at 120°, while Dana and Maffione stated that it occurs at 140°. In addition to all these factors, the particles' properties may also have a significant influence on the measurements. Properties such as particles size and color may contribute in deviating the measurement of turbidity, even when the TSS capacity of the samples is similar. This is because different particles properties may lead the incident light to be scattered in different directions or absorbed with different intensity. The usage of NIR (Near Infra Red) light sources (wavelength at 860 nm) can very much minimize the limitation related to colored particles.

Water quality fiber sensors presented in this paper introduced an innovative and novel approach for the water turbidity measurement. The results of measurement are compared with typical design of water turbidity measurement developed by Mohd Zubir and Bashah [50] and Daraigan [10]. The designs by Mohd Zubir and Bashah [50] and Daraigan [10] have shown almost a similar pattern of results, mainly due to the selection of optical components and parameters of the same type. The selection of very high intensity of light source and a very sensitive light detector have contributed significantly in improving the range of water turbidity measurements. These optical parameters were selected to fulfill the requirement of the application of optical fiber cable in the system which requires high capability optical components to be coupled with them in order to make the detection process work. The application of fiber optic in spectroscopic analysis provides the ability of “*taking the spectrometer to the sample*” despite the conventional method of taking the sample to the spectrometer [57]. Moreover, the fiber optic probe allows flexible delivery and collection of scattered or reflected light even in hard to reach areas [58]. The ultimate goal of the design of water quality fiber sensors is to make them able to be implemented at site where the consideration of the velocity of flowing media and the variation of particles may be considered and good measurement can still be obtained. Further modifications is therefore required before this desire can be translated into precision in measurements.

**Figure 11.**
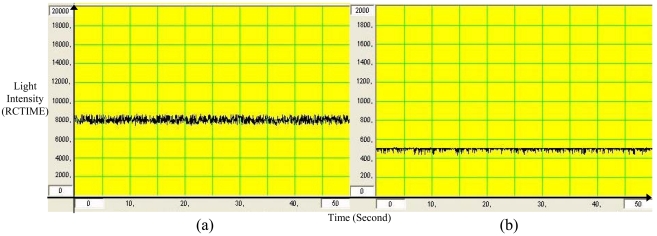
Stamp Plot Graph for Reverse Osmosis Water Collected by BLUE System through (a) Backscattered Measurement (b) Transmittance Measurement.

The major task in designing turbidimeters is to be able to measure the lowest amount of turbidity and manage to obtain similar results with high accuracy and precision when the measurement is performed repeatedly. This however may not be the same for the implementation of optical sensor in the measurement of turbidity or particles concentration in the flowing media which often desires the measurement of a very high capacity of suspended particles. The sensitivity of the system is indicated by the slope (multiplier of TSS) of the calibration equation. The slope of the graph is the indication of the intensity of detected scattered light in relative to the resolution of the measured sample. Higher slope is desirable here, since it shows that better resolution of TSS can be measured (smaller capacity of TSS). Results of the measurements were expected to be in linear correlation between the light detected in the unit of RCTIME and the amount of TSS. However, the graph will move away from its linear line once the range of turbidity measured is at higher concentrations. This is due to the nature of turbidity itself that is non linear with the intensity of scattered light and also due to the nature of the system which actually responds in exponential form to the light intensity (refer to Figure 9a,b). For every individual sample measured, the level of measured light signal was actually fluctuating at constant rate. This can be seen through graph plotted by Stamp Plot software which shows the behavior of light intensity (RCTIME) when measured continuously for reverse osmosis water (clear water). A steady result of RCTIME is needed to represent the amount of TSS or level of turbidity in any sample measured. Therefore, a mathematical manipulation has been performed on the set of RCTIME measured to represent the value of sample turbidity. Here, the first 50 data was collected by the Basic Stamp 2 Microcontroller and the highest RCTIME was chosen to represent the level of TSS of the sample. This was made due to the understanding that the pattern of light intensity fluctuation tends to differ between high and low RCTIME as shown in Figure 11a,b. This mathematical manipulation technique is seen as the best resolution that suits any level of turbidity measured. However, the fluctuation of RCTIME still exists but within minimized range. To be able to further minimize this fluctuation can lead to the improvement of R^2^ for the calibration of linear equation which will also reduce the RMSE and improve the resolution of turbidity measurement.

## 12. Summary

In the development of optical fiber sensors for water turbidity measurements, there are multiple constraints that need to be emphasized before reliable and precise measurements can be produced. On the instrumentations side, the selection of optical components (light sources and detectors) and the developed measuring techniques is essentials. The combinations between the light source and detector define the spectral characteristics of the turbidimeter and the behavior of its respond to the sample. The maximum efficiency of the system can be obtained by matching well the source and the detector, especially on their spectral response. 90° measuring techniques are commonly being used in water and wastewater analysis due to their lesser sensitivity to variations in particles size. The increase in optical path length may increase the sensitivity of an instrument, but it may affect its linearity at high particle concentrations due to the existence of multiple scattering. Particles properties such as size, color, shape and composition are factors which may influence the value of turbidity and its correlation with the capacity of suspended particles in water. These factors may influence the direction of scattered light and the intensity of light which will be absorbed by the particles. Different sizes of particles may cause the light to be scattered with different intensity for different incident light wavelengths. Besides, it may also dictate the different settling time for particles which may as well influence the measurement of turbidity and capacity of suspended particles whether in the measurement chamber (in laboratory) or at field. The development of optical fiber sensors by Omar and Matjafri [51] present promising techniques for the flexible turbidity measurements. The small sized optical systems with small probes make the systems highly mobile for field measurements. The systems which currently have been successfully developed and tested within the lab need to undergo further modifications to make them suitable for field measurements. The current systems with exponential response curve need to be linearized through the application of analogue to digital converter, instead of the usage of RCTIME. This is to enhance the full scale measurement of the systems since the current systems decreases in its resolution at high concentration of particles. The systems need to be equipped with stray light elimination ability since the current implementations were conducted in dark chambers. Further experiments need to be conducted on particles with different properties and extensive analysis need to be taken in deriving algorithm on the relationship of all these factors to the measurement of turbidity or concentration of particles towards the intensity of measured signal through optical fiber sensors. With all these in hand, it is hope that the newly develop systems will possess higher reliability and precision to be implemented in dynamic environmental conditions.

## 13. Conclusions

This paper has presented the overall design concept and factors to be put forward as major considerations in the development of optical instruments for water turbidity measurement. This paper has also shown a design which can be developed through a very simple circuitry which can be implemented by school level students, as has been designed by Mohd Zubir and Bashah [50]. However, this is achieved by compromising the resolution of the measurement. A more comprehensive design and experiments were conducted by Daraigan [10]. Water quality fiber sensors have successfully introduced an innovative approach for water turbidity measurement with higher sensitivity and reliability, but further effort is in progress to make the system able to be implemented *in situ*.

## Figures and Tables

**Table 1.** Relationship between Turbidity and TSS conducted by four researchers [9-12].

**Researcher**	**Sample**	**Relationship**	**R^2^**	**Range of TSS (mg/L) Measured**
Holliday *et al.* [9]	Clay	NTU = 0.7733(TSS)^0.9336^	0.9996	0–1,000
	Silt + Clay	NTU = 1.0283(TSS)^1.0282^	0.9991	0–1,000
	Whole Soil	NTU = 0.4833(TSS)^1.012^	0.9987	0–1,000
Daraigan [10]	Silt + Clay	NTU = 1.1595(TSS)^0.9389^	0.9873	0–1,000
Omar and MatJafri [11]	Clay	NTU = 0.7991(TSS)	0.9906	0–100
	Rust	NTU = 0.9729(TSS)	0.9927	0–100
Baker *et al.* [12]	1.47μm Clay	NTU = 1.25(TSS)	0.99	0–35
	5.1μm Clay	NTU = 0.52(TSS)	0.98	0–35
	15.9μm Clay	NTU = 0.19(TSS)	0.99	0–35
	22.9μm Clay	NTU = 0.14(TSS)	0.99	0–35
	28.6μm Clay	NTU = 0.011(TSS)	0.99	0–35

**Table 2.** Correlation Coefficient (R^2^) and Root Mean Square Error (RMSE) for Different Angle of Scattering Light [10].

**58°**		**71°**		**78°**		**90°**		**180°**	
R^2^	RMSE (mg/L)	R^2^	RMSE (mg/L)	R^2^	RMSE (mg/L)	R^2^	RMSE (mg/L)	R^2^	RMSE (mg/L)
0.975	20.463	0.948	29.476	0.827	53.672	0.979	18.892	0.933	33.439

**Table 3.** Correlation Coefficient (R^2^) and Root Mean Square Error (RMSE) for the measurement of scattering, absorption and reflected light with different wavelength of light sources [10].

**Techniques**	**Wavelength** **(λ=nm)**	**R^2^**	**RMSE** **(mg/L)**
Scattering Measurement (90° Scattering – D1)	950	0.882	62.005
	875	0.921	50.617
	635	0.765	92.741
	950 and 875	0.957	39.699
	950 and 635	0.912	56.697
	875 and 635	0.952	42.024
	950, 875 and 635	0.912	60.608
Absorption Measurement (Transmittance / 0° Scattering – D2)	950	0.878	62.827
	875	0.806	78.898
	635	0.916	52.109
	950 and 875	0.899	60.425
	950 and 635	0.967	37.124
	875 and 635	0.945	44.993
	950, 875 and 635	0.962	39.692
Surface Reflection Measurement	950	0.999	10.093
	880	0.961	54.841
	950 and 880	0.997	15.206

**Table 4.** Optical Components and Parameters [51].

**Components and Parameters**	**BLUE System**	**RED System**
**LED**	**Blue (Part No. E1L51-3B0A2-02)**	**Red (Part No. HLMP-EG08-WZ000)**
Typical Wavelength	470 nm	635 nm
Maximum Intensity	4860 mcd	16000 mcd
Typical Viewing Angle	15°	6°
DC Forward Current	30mA	50mA
Typical Forward Voltage	3.4V	1.9V
**Photodiode**	**Blue (470 nm) TSLB257**	**Red (635 nm) TSLR257**
Irradiance Responsivity	1.7 μW/cm^2^ (approx. 11.611 lux)	1.1 μW/cm^2^ (approx. 7.513 lux)
Typical Output Voltage	2 Volt	2 Volt

**Table 5.** BLUE and RED System Response Equation.

	**BLUE System**	**RED System**
**System Response**	y = 4×10^6^ (e^-2.1687×^)	y = 4×10^6^ (e^- 2.915×^)
**R^2^**	0.9848	0.9663

**Table 6.** Comparison between Results Measured by BLUE and RED Systems.

	**BLUE System**	**RED System**

**Backscattering:**		

**Calibration:**		
Response - Linear Equation	RCTIME = −21.775 (TSS) + 7841.6	RCTIME = −9.6064 (TSS) + 3092.8
RMSE (mg/L)	12.99	23.22
R^2^	0.958	0.867

**Validity Test** **(measured vs estimated):**		
**Terengganu River**:		
Linear Relationship	y = 0.9662x	y = 0.9708x
R^2^	0.945	0.949
**House Water Supply**:		
Linear Relationship	y = 1.0358x	y = 1.0039x
R^2^	0.7512	0.9573

**Transmittance (0° Scattering):**		

**Calibration:**		
Response–Linear Equation	RCTIME = 2.7969 (TSS) + 429.77	RCTIME = 0.8025 (TSS) + 295.94
RMSE (mg/L)	13.38	11.95
R^2^	0.9889	0.9912

**Turbidity Measurement at Endau Rompin National Park** **(calibrated in NTU)**		
Linear Relationship	RCTIME = 2.0811 (NTU) + 479.22	RCTIME = 1.5562 (NTU) + 271.43
RMSE (NTU)	2.206874	1.58928
R^2^	0.8747	0.935

**90° Scattering:**		

**Calibration:**		
Response–Linear Equation	RCTIME = −43.369 (TSS) + 33915	RCTIME = −82.094 (TSS) + 28454
RMSE (mg/L)	7.79	4.43
R^2^	0.9412	0.9809
